# Comprehensive Analysis of m^5^C RNA Methylation Regulator Genes in Clear Cell Renal Cell Carcinoma

**DOI:** 10.1155/2021/3803724

**Published:** 2021-09-28

**Authors:** Jiajin Wu, Chao Hou, Yuhao Wang, Zhongyuan Wang, Pu Li, Zengjun Wang

**Affiliations:** The First Affiliated Hospital of Nanjing Medical University, No. 300 Guangzhou Road, Nanjing 210029, China

## Abstract

**Background:**

Recent research found that N5-methylcytosine (m^5^C) was involved in the development and occurrence of numerous cancers. However, the function and mechanism of m^5^C RNA methylation regulators in clear cell renal cell carcinoma (ccRCC) remains undiscovered. This study is aimed at investigating the predictive and clinical value of these m^5^C-related genes in ccRCC.

**Methods:**

Based on The Cancer Genome Atlas (TCGA) database, the expression patterns of twelve m^5^C regulators and matched clinicopathological characteristics were downloaded and analyzed. To reveal the relationships between the expression levels of m^5^C-related genes and the prognosis value in ccRCC, consensus clustering analysis was carried out. By univariate Cox analysis and last absolute shrinkage and selection operator (LASSO) Cox regression algorithm, a m^5^C-related risk signature was constructed in the training group and further validated in the testing group and the entire cohort. Then, the predictive ability of survival of this m^5^C-related risk signature was analyzed by Cox regression analysis and nomogram. Functional annotation and single-sample Gene Set Enrichment Analysis (ssGSEA) were applied to further explore the biological function and potential signaling pathways. Furthermore, we performed qRT-PCR experiments and measured global m^5^C RNA methylation level to validate this signature in vitro and tissue samples.

**Results:**

In the TCGA-KIRC cohort, we found significant differences in the expression of m^5^C RNA methylation-related genes between ccRCC tissues and normal kidney tissues. Consensus cluster analysis was conducted to separate patients into two m^5^C RNA methylation subtypes. Significantly better outcomes were observed in ccRCC patients in cluster 1 than in cluster 2. m^5^C RNA methylation-related risk score was calculated to evaluate the prognosis of ccRCC patients by seven screened m^5^C RNA methylation regulators (NOP2, NSUN2, NSUN3, NSUN4, NSUN5, TET2, and DNMT3B) in the training cohort. The AUC for the 1-, 2-, and 3-year survival in the training cohort were 0.792, 0.675, and 0.709, respectively, indicating that the risk signature had an excellent prognosis prediction in ccRCC. Additionally, univariate and multivariate Cox regression analyses revealed that the risk signature could be an independent prognostic factor in ccRCC. The results of ssGSEA suggested that the immune cells with different infiltration degrees between the high-risk and low-risk groups were T cells including follicular helper T cells, Th1_cells, Th2_cells, and CD8+_T_cells, and the main differences in immune-related functions between the two groups were the interferon response and T cell costimulation. In addition, qRT-PCR experiments confirmed our results in renal cell lines and tissue samples.

**Conclusions:**

According to the seven selected regulatory factors of m^5^C RNA methylation, a risk signature associated with m^5^C methylation that can independently predict prognosis in patients with ccRCC was developed and further verified the predictive efficiency.

## 1. Introduction

Renal cell carcinoma (RCC) accounts for approximately 90% of all kidney tumors [1].

Clear cell renal cell carcinoma (ccRCC) is the most common histological subtype of RCC, accounting for 70% [2]. Surgical treatment is still an effective way to treat ccRCC, because it is not sensitive to radiotherapy and chemotherapy [3, 4]. However, nearly a third of patients were reported experiencing a recurrence after surgery with a median survival time of 1.9 years, resulting in a worse overall survival (OS) [5]. Due to the unclear biological progression and molecular mechanism of ccRCC, it is difficult to predict the prognosis of patients with ccRCC accurately. Therefore, we urgently need to find the potential developmental mechanisms of ccRCC and search for new therapeutic targets with better diagnostic and prognostic value.

RNA methylation accounts for more than 60 percent of all RNA modifications, where N5-methylcytosine (m^5^C) RNA methylation is the second common type, only inferior to N6-methyladenosine (m^6^A) methylation [6]. m^5^C RNA methylation, discovered in 1925, is widely distributed in various types of RNA methylation, including messenger RNA (mRNA), transport RNA (tRNA), enhancer RNA (eRNA), and ribosomal RNA (rRNA) [7, 8]. Recent studies revealed that 5-methylcytosine (m^5^C) methylation modification of the transcriptomes, mainly concentrated in 3′ untranslated regions (3′ UTRs), played an essential role in the regulations of mRNA such as its splicing translation and stability and is even involved in the interactions between RNA and protein [9–11]. The methyltransferase complex of m^5^C was composed of “writers” (NSUN2, NSUN3, NSUN4, NSUN5, NSUN7, NOP2, DNMT1, TRDMT1, DNMT3A, and DNMT3B) that, respectively, install and reverse the methylation; “eraser” (TET2) that is a demethylase and RNA-binding protein; and “readers” (ALYREF) that recognize mRNA m^5^C sites [10, 12–14].

Recently, increasing studies have suggested that m^5^C methylation also plays a vital role in pathological conditions such as cancer [15]. Chen et al. revealed that m^5^C methylation could promote the pathogenesis of human bladder urothelial carcinoma by stabilizing oncogene mRNAs [16]. Another study indicated that gene signatures of m^5^C methylation genes might predict prognoses of patients with head and neck squamous cell carcinoma [9]. However, the potential mechanisms of m^5^C RNA modification involved in the development of ccRCC were still unknown.

In this present research, we aim to explore the association between ccRCC patients with the expression of RNA m^5^C modification genes in the TCGA-KIRC cohort. Furthermore, the relationships between the genes and the clinicopathological parameters were also analyzed. Then, we screened out seven m^5^C RNA methylation-related genes to construct a risk signature model to predict the overall survival (OS) of the patients with ccRCC. The prediction accuracy of this risk signature was further validated in the testing group and the entire cohort, respectively.

## 2. Materials and Methods

### 2.1. Data Acquisition and Preprocessing

All public gene expression data, along with their clinical annotations such as age, gender, grade, stage, TNM classification, and survival status, were acquired from TCGA (http://cancergenome.nih.gov/). The gene expression profiles of 539 ccRCC cases and 72 normal controls were analyzed for further research, and 513 ccRCC patients with complete clinical information, including OS, were further selected from the 539 ccRCC cases (Table 1).

### 2.2. Identification of Differentially Expressed m^5^C-Related Genes in TCGA Database

We screened differential expression genes (DEGs) according to the adjusted *p* value < 0.05, log_2_ fold change > 1 or < −1 between the ccRCC tissue samples and normal renal tissue samples. The m^5^C RNA methylation regulator genes were further screened from DEGs. Univariate Cox regression analysis was used to evaluate the m^5^C RNA methylation regulator genes significantly related to OS according to *p* value < 0.05.

### 2.3. Construction of PPI Network and Correlation Analysis

Protein–protein interaction (PPI) network was constructed for these twelve m^5^C methylation regulators using the STRING online database (http://string-db.org/). Moreover, by using the Pearson correlation analysis, we performed the correlation analysis among these regulators and the different clinicopathological parameters were also analyzed based on the TCGA database. Wilcoxon's test compared the diverse expression of m^5^C RNA methylation regulators between ccRCC samples and normal samples.

### 2.4. Consensus Clustering Analysis to Define m^5^C Subtypes

To reveal the relationships between the expression levels of m^5^C-related genes and the prognosis of ccRCC, the tumor samples were clustered into different groups with the R package “Consensus Cluster Plus.” To verify different gene expression patterns in different ccRCC groups, principal component analysis (PCA) was further performed. We performed Kaplan-Meier survival analysis to reveal the differences in OS between different clusters. Additionally, we applied the Chi-square test for comparing the clinicopathological parameters including gender, grade, age, TNM stage, and stage between different clusters.

### 2.5. m^5^C-Related Prognostic Signature Generation and Prediction

Univariate cox analysis was carried out to identify possible prognostic m^5^C RNA methylation regulators in ccRCC. Then, m^5^C RNA methylation-related risk score was constructed by using the LASSO Cox Regression algorithm in the training cohort. The m^5^C-related risk signature was calculated with the following formula:

(1) $$\begin{array}{c} \text{Risk score}=\overset{n}{\underset{i=1}{\sum }}\text{c}\text{oef}\left(i\right)\times x\left(i\right), \end{array}$$

where *n*, coef(*i*), and *x*(*i*) represent the number of genes, coefficient, and the relative expression value of each gene, respectively. On the basis of the median score in the training cohort, we divided all ccRCC patients into the low-risk and high-risk groups. Log-rank test and Kaplan-Meier curve were applied, respectively, for revealing whether the risk score can differentiate the OS in ccRCC patients. To evaluate the predictive ability of these risk model, we further analyzed the receiver operating characteristic (ROC) curve and the area under the ROC curve (AUC) by the package of “survivalROC” in R language.

### 2.6. Validations of the Seven-Gene Risk Score in the Testing and the Entire Cohort

The seven-gene risk score was further verified in the testing and entire cohort. Log-rank test and Kaplan-Meier curve were applied, respectively, for revealing whether the risk score can differentiate the OS in ccRCC patients. ROC and AUC were further analyzed in the testing and entire cohort.

### 2.7. Construction and Validation of Nomogram

The nomogram was applied to predict survival of ccRCC patients. Selected seven m^5^C RNA methylation regulators genes and survival states were used to build the nomogram using R “rms” packages. The calibration curve was used to evaluate the accuracy of the nomogram in differentiating between patient groups.

### 2.8. GO and KEGG Pathway Enrichment Analyses

GO and KEGG enrichment analyses of seven selected genes were performed with R packages “clusterProfiler,” “enrichplot,” and “ggplot2.” Only terms with both *p* and *q* values of < 0.05 were considered significantly enriched.

### 2.9. Single-Sample Gene Set Enrichment Analysis (ssGSEA)

To further evaluate the prognostic value of this risk model in TCGA, single-sample Gene set enrichment analysis (ssGSEA) was performed in two different risk score groups. When the false detection rate (FDR) was less than 0.25 and the normalized *p* value was less than 0.05, it was considered to be significantly enriched.

### 2.10. Clinical Tissue Samples

We gathered clear cell renal cell carcinoma (ccRCC) tumors and adjacent normal tissue samples from patients who had undergone radical nephrectomy surgery in The First Affiliated Hospital of Nanjing Medical University between January 2003 and March 2019. This study was ethically authorized by Ethics Committees of the First Affiliated Hospital of Nanjing Medical University. All the patients signed the agreement for permission that their tissue samples and other clinical information may be used for further research purposes.

### 2.11. Cell Culture

The renal cancer cell lines (786-O, Caki-1) and the human renal tubular epithelial immortalized cell line (HK-2) were purchased from the Type Culture Collection of the Chinese Academy of Sciences (Shanghai, China) and cultured in RPMI 1640 (786-O); McCoy's 5A (Caki-1) and DMEM/F12 (HK-2) (Gibco, Thermo Fisher Scientific, USA) containing 10% fetal bovine serum (FBS; Gibco, Thermo Fisher Scientific, USA) and 1% penicillin/streptomycin (Gibco, Thermo Fisher Scientific, USA). All cell lines were cultured at 37°C in a humidified incubator containing 5% CO_2_.

### 2.12. Total RNA Isolation and qRT-PCR

Total RNA was extracted from cultured cell lines and tissue samples using TRIzol reagent (Thermo Fisher Scientific, USA) and subsequently reverse transcribed into cDNA using PrimeScript RT reagent (Takara, Japan), according to the manufacturer's instructions. qRT-PCR experiment was performed with SYBR Premix Ex Taq Reagent (Takara, Japan) using the StepOne Plus Real-Time PCR system (Applied Biosystems, USA). The primers used for qRT-PCR were listed in Table S1. The mRNA expression level was calculated using the 2^−*ΔΔ*Ct^ method and normalized against *β*-actin with ABI StepOne software version 2.1.

### 2.13. Determination of Total m^5^C RNA Modification Level

Total m^5^C RNA modification level was detected in 200 ng of total RNA extracted from cells using the EpiQuik Global RNA Methylation Assay Kit (5 Methyl Cytosine, Fluorometric) (Abcam, USA) according to the manufacturer's protocols. Briefly, m^5^C in RNA is detected using capture and detection antibodies and then quantified fluorometrically by reading the fluorescence in a microplate spectrophotometer. The detected signal was calculated by reading wavelength of 530/590 nm using a microplate reader. The experiments were performed in triplicate.

### 2.14. Statistical Analysis

All statistical data and figures were analyzed by R 4.0.3. Using Wilcoxon's test, we assessed the different expressions of m^5^C RNA methylation regulators between ccRCC patients and normal tissues. The correlation analysis among m^5^C RNA methylation regulators was carried out by the Pearson correlation analysis. The chi-square test was performed to evaluate the association between the risk score and clinicopathological parameters. All statistical results with *p* < 0.05 were considered statistically significant.

## 3. Results

### 3.1. Screening Differently Expressed m^5^C Methylation Regulator Genes in ccRCC

**Figure 1:**
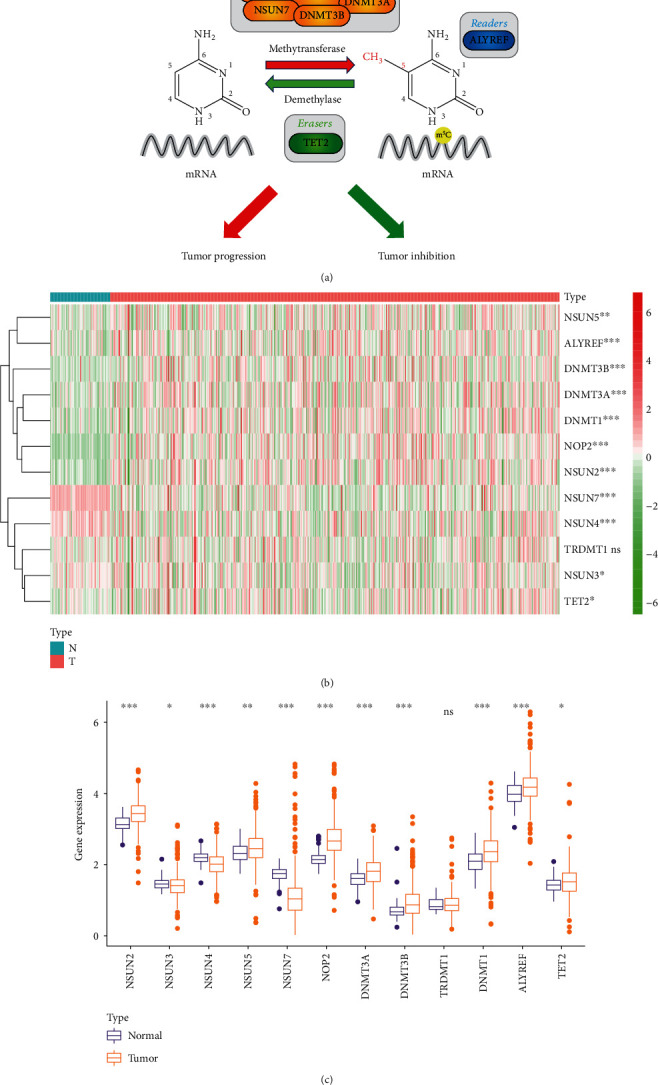
Different expression of m^5^C RNA methylation-related genes in ccRCC. (a) Schematic flow chart of N5-methylcytosine (m^5^C) RNA methylation. (b) The heatmap of twelve m^5^C RNA methylation regulators in 539 ccRCC and 72 normal tissues from the TCGA database. The color bar from red to green denotes high to low gene expression. (c) The expression of twelve m^5^C RNA methylation regulators in normal tissues and ccRCC from the TCGA database. ^∗^*p* < 0.05, ^∗∗^*p* < 0.01, and ^∗∗∗^*p* < 0.001. ns: no significance.

According to the previous studies [10–12], we analyzed 12 m^5^C RNA methylation-related genes, including NSUN5, ALYREF, DNMT3B, DNMT3A, NSUN2, NOP2, DNMT1, NSUN3, NSUN4, NSUN7, TET2, and TRDMT1. Firstly, we learned the mechanism of N5-methylcytosine (m^5^C) RNA methylation, and the sketch map was shown in Figure 1(a). We analyzed the mRNA expression levels of 12 m^5^C RNA methylation genes in renal clear cell carcinoma (ccRCC) (*n* = 539) and normal samples (*n* = 72) obtained from the TCGA-KIRC database. The heatmap indicating the expression levels of m^5^C regulatory genes showed that 12 m^5^C RNA methylation regulators were differently expressed in ccRCC tissues compared with normal tissues (Figure 1(b)). As shown in the boxplot, NSUN5, ALYREF, DNMT3B, DNMT3A, NSUN2, NOP2, and DNMT1 were upregulated, while NSUN3, NSUN4, NSUN7, and TET2 were downregulated (Figure 1(c)). Besides, the expression level of 12 m^5^C-related genes in various tumors obtained from the TCGA database was also analyzed (Figure S1).

### 3.2. Functional Annotation and Pathway Enrichment Analyses of Twelve m^5^C-Related Genes

**Figure 2:**
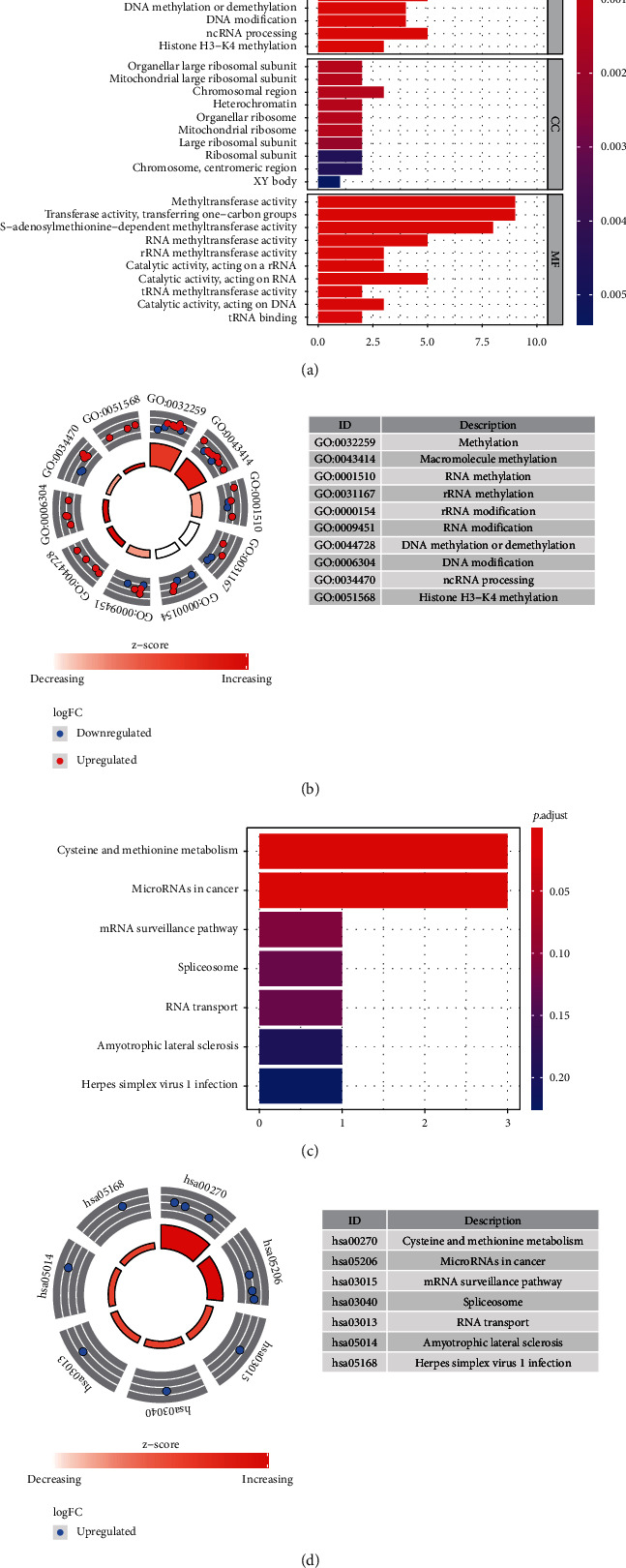
Enrichment plots of twelve m^5^C RNA methylation-related genes by performing GSEA. (a) GO enrichment analysis of twelve m^5^C RNA methylation-related genes in the TCGA-KIRC cohort. (b) KEGG pathway analysis of twelve m^5^C RNA methylation-related genes in the TCGA-KIRC cohort.

To identify the function and potential pathway involved in ccRCC of m^5^C RNA methylation genes, we perform GO and KEGG enrichment analyses. Our results revealed that these m^5^C RNA methylation genes were remarkably enriched in the biological processes (BP) associated with methylation, macromolecule methylation, RNA methylation, and RNA modification. Among the molecular function (MF) analyses, they were significantly enriched in chromosomal region, heterochromatin, and organellar large ribosomal subunit. Through the cellular component (CC), m^5^C RNA methylation genes were significantly enriched in methyltransferase activity, methyltransferase activity, RNA methyltransferase activity, and rRNA methyltransferase activity (Figures 2(a) and 2(b)).

KEGG pathway analysis found that m^5^C RNA methylation genes were mainly enriched in cysteine and methionine metabolism, microRNAs in cancer, mRNA surveillance, spliceosome, and RNA transport (Figures 2(c) and 2(d)).

### 3.3. Construction of a PPI Network and Correlation Analysis

**Figure 3:**
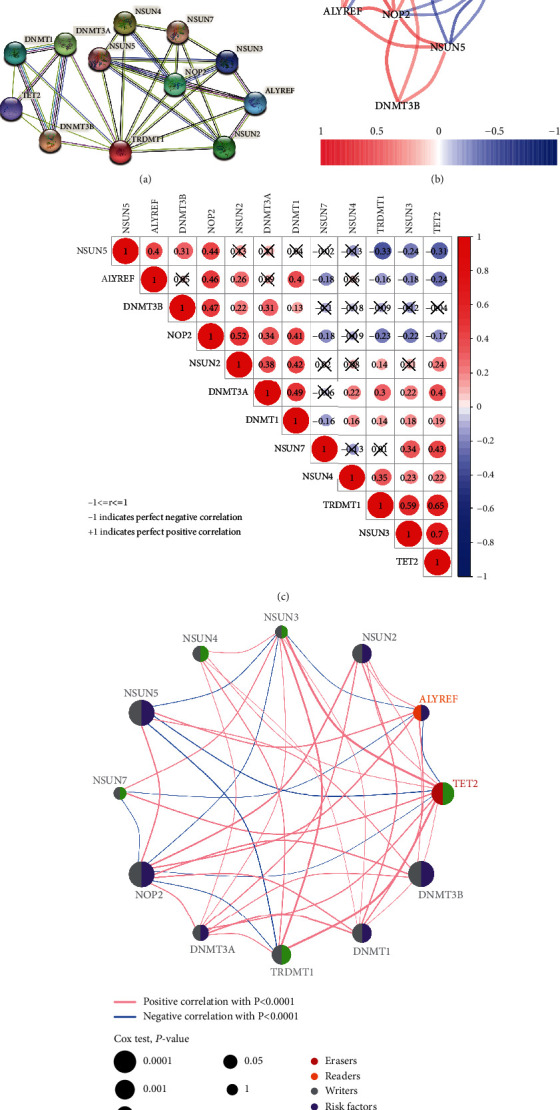
PPI network and correlation analyses of m^5^C RNA methylation-related genes. (a) PPI network of the eleven differentially expressed m^5^C RNA methylation regulatory genes. (b) Correlation analysis at the transcriptional level. (c) Pearson's correlation analysis of these m^5^C RNA methylation-related genes in the TCGA. Note: ‘*r*' denotes Pearson's correlation coefficient whose value ranges between -1 (perfect negative correlation) and +1 (perfect positive correlation). (d) A network plot of the function and correlation analysis by Cox test.

To further explore the underlying mechanism, the PPI network was constructed on the basis of the STRING database. PPI network analysis showed that NSUN5, NSUN3, NSUN4, NSUN7, NOP2, and TET2 were the essential genes (Figure 3(a)). In correlation analysis, we found that these genes were strongly correlated at the transcriptional level (Figure 3(b)). Among the regulatory factors of m^5^C RNA methylation, the positive interaction between NSUN3 and TET2 (r = 0.7) and the negative interaction between NSUN5 and TET2 (*r* = −0.31) were the most significant (Figure 3(c)). In addition, a network diagram showed the interaction, function, and prognostic value between twelve m^5^C RNA methylation regulators (Figure 3(d)).

### 3.4. Genetic Alterations of m^5^C RNA Methylation Regulators and Association with Immune Cell Infiltration

**Figure 4:**
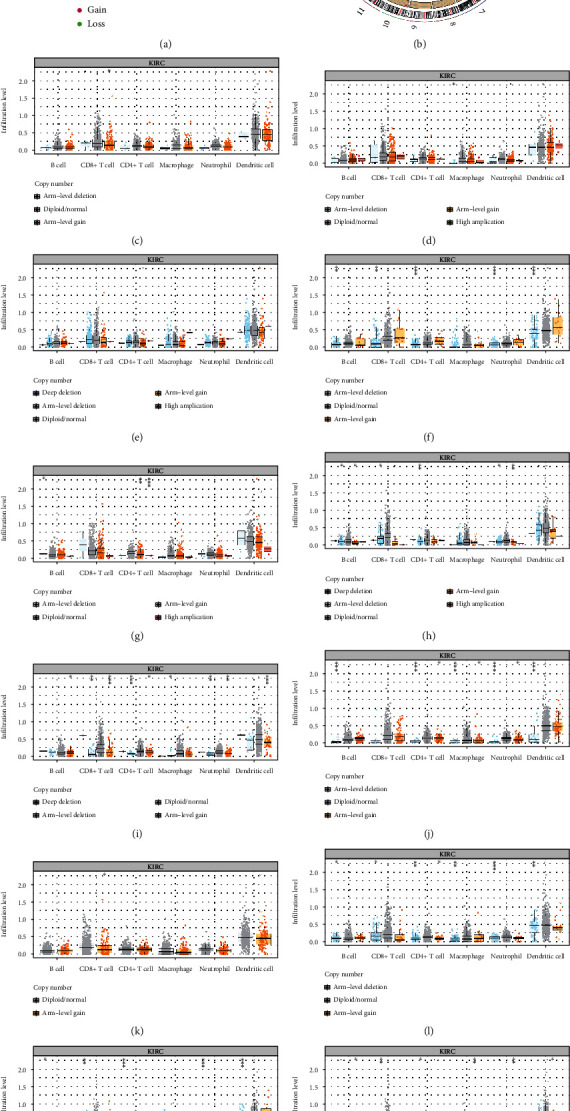
Correlations between CNV and immune cell infiltrations of twelve m^5^C modification regulators in ccRCC. (a) The copy number variation (CNV) frequency percentage of m^5^C regulators in ccRCC. The red dot represents the CNV amplification, and the green dot represents the CNV deletion. (b) The location of CNV of m^5^C regulators on chromosomes. (c–n) Correlation analysis between the CNV of m^5^C-related signature and immune cell infiltration. (c) NOP2. (d) NSUN2. (e) NSUN3. (f) NSUN4. (g) NSUN5. (h) NSUN7. (i) DNMT1. (j) DNMT3A. (k) DNMT3B. (l) TRDMT1. (m) ALYREF. (n) TET2. ^∗^*p* < 0.05, ^∗∗^*p* < 0.01, and ^∗∗∗^*p* < 0.001. ns: no significance.

Then, we investigate the relationship between twelve m^5^C RNA methylation regulators and CNV mutations. We observed widespread CNV on twelve m^5^C RNA methylation regulators through copy number variation (CNV) analysis. Among these genes, NOP2, ALYREF, NSUN2/3, and TRDMT1 showed high CNV amplification frequency. In contrast, NSUN4/5/7, TET2, and DNMT1/3A/3B had significantly high CNV deletion frequency (Figure 4(a)). The locations of CNV of twelve m^5^C RNA methylation regulators on chromosomes are shown in Figure 4(b). Nevertheless, the effects of CNV of the m^5^C RNA methylation regulators-based signatures were further analyzed to clarify the association with different immune cell infiltrations. Adopting the TIMER database, the CNV of the identified m^5^C regulators signatures, including deep deletion, arm-level deletion, diploid/normal, arm-level gain, and high amplification, significantly affected the infiltration levels of B cells, CD4+ T cells, CD8+ T cells, neutrophils, macrophages, and dendritic cells in ccRCC (Figures 4(c)–4(n)). These results illustrated the underlying mechanisms that m^5^C RNA methylation regulators had pivotal regulatory effects on the tumor immune microenvironment for ccRCC patients.

### 3.5. Consensus Cluster Analysis to Define m^5^C Subtypes

**Figure 5:**
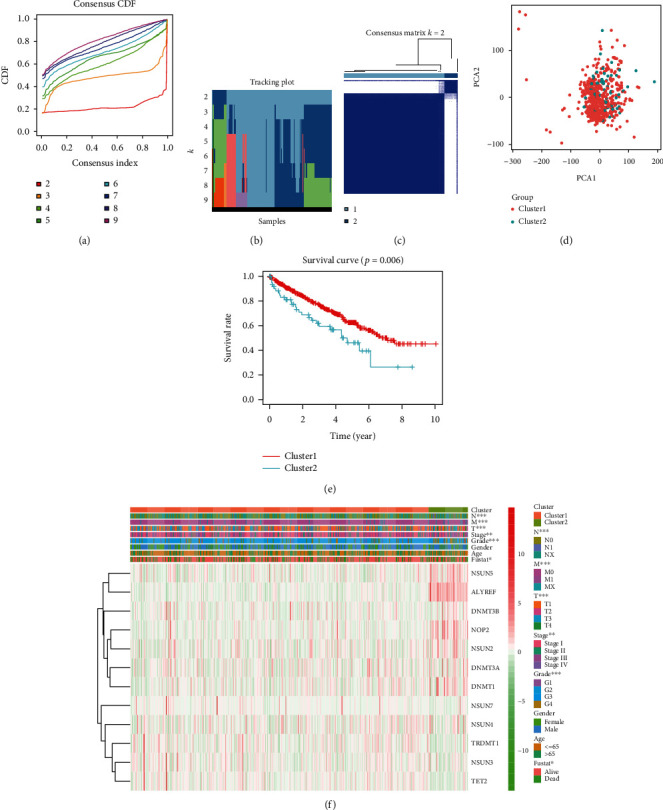
Consensus clustering analysis shows two clusters of ccRCC patients with differential prognosis. (a) Cumulative distribution function (CDF) curves for the consensus score (*k* = 2 to 9). (b) The tracking plot for *k* = 2 to 9. (c) Consensus clustering matrix for the optimal cluster number, *k* = 2. (d) Principal component analysis of the total RNA expression profile. ccRCC in cluster 1 and 2 are marked in red and blue, respectively. (e) Kaplan-Meier overall survival (OS) curve for ccRCC patients in cluster 1 and 2. (f) The expression heatmap of the 12 m^5^C methylation regulatory genes in cluster 1 and cluster 2 patients that were stratified according to the clinicopathological parameters: namely, N stage (N0, N1, or NX), M stage (M0, M1, or MX), T stage (T1-T4), AJCC stages (stages I, II, III ,or IV), grade (G1-G4), gender (male or female), age (>65 y or <65 y), and survival status (alive or dead). ^∗^*p* < 0.05, ^∗∗^*p* < 0.01, and ^∗∗∗^*p* < 0.001.

We used unsupervised consensus clustering analysis to identify two subtypes based on the m^5^C RNA methylation regulator expression profiles. Clearly, *k* = 2 was assumed to be the most appropriate choice for dividing the tumor samples into two different clusters (Figures 5(a)–5(c)). Additionally, the principal component analysis (PCA) was performed to compare the transcriptional profile between the two clusters, and the result was shown in Figure 5(d). For comparing the overall survival (OS) of ccRCC patients between the two clusters, the Kaplan–Meier method was applied. The result indicated that the ccRCC patients in cluster 1 had a significantly longer OS than cluster 2 (*p* = 0.006) (Figure 5(e)). The comparison of clinicopathological features between the two clusters indicated a significant difference in terms of the stage (*p* < 0.01), grade (*p* < 0.001), and TNM stage (*p* < 0.001), while there was no significant difference in age and gender between the two clusters (Figure 5(f)). The clinicopathological features between two clusters were shown in Table 2. In conclusion, the results above suggested that the clustering was intimately connected with the clinicopathological characteristics of ccRCC.

### 3.6. Prognostic Risk Signature of m^5^C RNA Methylation Regulators

**Figure 6:**
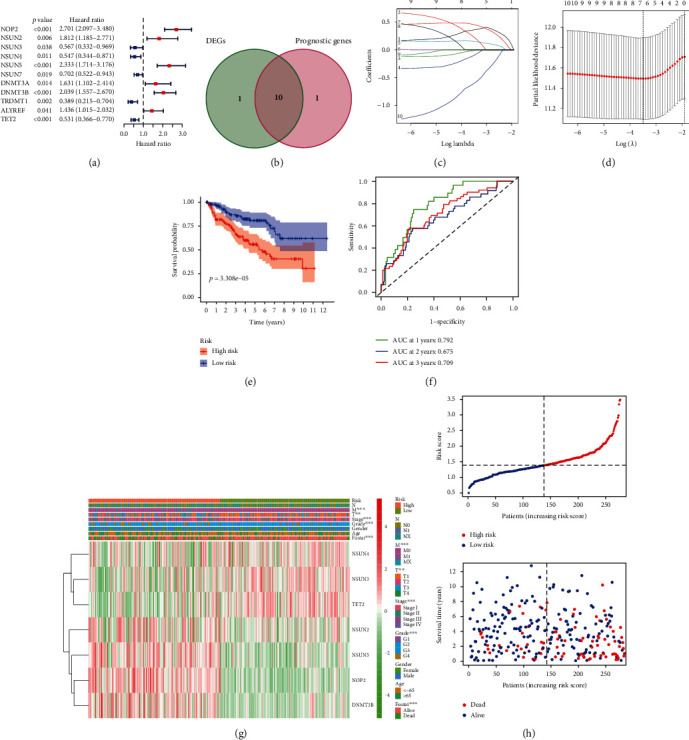
Construction and evaluation of the m^5^C RNA methylation-related prognostic risk signature in the training cohort. (a) Univariate Cox regression analysis results show the *p* values and hazard ratios (HR) with confidence intervals (CI) of the twelve m^5^C RNA methylation regulatory genes. (b) The Venn diagram between differentially expressed genes and genes related to OS. (c, d) The 7 prognostic risk signature genes were selected by LASSO Cox regression analysis. (e) Kaplan-Meier survival curves show the overall survival (OS) rates of high-risk (*n* = 135) and low-risk (*n* = 132) ccRCC patients of the training cohort. The high-risk group shows shorter OS compared to the low-risk group. (f) The accuracy and reliability of the prognostic risk signature in determining the 1-year, 2-year, and 3-year survival outcomes of the high- and low-risk patients in the training cohort. (g) The expression heatmap of the seven prognostic risk-related m^5^C RNA methylation regulators in the high-risk (blue) and low-risk (pink) ccRCC patients of the training cohort. ^∗∗^*p* < 0.01; ^∗∗∗^*p* < 0.001. (h) The distributions of risk scores of the high-risk (red) and low-risk (blue) ccRCC patients and corresponding survival time (the red dots represent the dead patients and the blue dots represent the alive patients) in the training cohort.

Then, we performed univariate Cox regression analysis based on the expression levels of these regulators from TCGA to investigate the prognostic value of these twelve m^5^C RNA methylation regulators in ccRCC. The results suggested that eleven regulators were significantly associated with overall survival (OS) (*p* < 0.05), among which, TET2, NSUN3, NSUN4, NSUN7, and TRDMT1 were considered as protective genes with *HR* < 1, while NSUN5, NSUN2, DNMT3B, DNMT3A, ALYREF, and NOP2 were considered as risky genes with *HR* > 1(Figure 6(a)). Furthermore, ten genes were intersected from differentially expressed genes and genes related to OS by the Venn diagram (Figure 6(b)). The LASSO Cox regression analysis, including these ten genes, was performed to predict the prognosis of ccRCC through m^5^C RNA methylation regulators better. Consequently, seven genes, composed of NOP2, DNMT3B, NSUN3, NSUN5, TET2, NSUN2, and NSUN4, were screened for the construction of a prognostic risk signature via LASSO Cox regression analysis (Figures 6(c) and 6(d)). The expression levels of the seven genes selected out in various cancers on the basis of the TCGA database were consistent with the result we obtained in ccRCC (Figure S1B-H). The risk score for each patient was calculated with the following formula (Table 3):

m^5^C Risk Signature = 0.358712202257608∗NOP2 + 0.229671617272167∗NSUN2 + 0.150827601647872∗NSUN5 + 0.453815651228113∗DNMT3B + (−0.00949630611801698)∗NSUN3 + (−0.604181882439325)∗TET2 + (−0.09915438936325)∗NSUN4.

For the sake of evaluating the prognostic value of the risk signature, 267 random selected ccRCC patients with complete follow-up information in the training cohort were divided into the high-risk group (135 patients) and the low-risk group (132 patients) according to the calculated median risk score above, and the overall survival of the two groups was compared. The result indicated that the low-risk ccRCC patients had a better prognosis with a longer OS (Figure 6(e)). The time-dependent ROC curve analysis suggested that the prognostic signature with the AUC values for the 1-, 2-, and 3-year survival in the training cohort were 0.792, 0.675, and 0.709, respectively (Figure 6(f)). The result of PCA in the training cohort was shown in Figure S5A, and the result of stochastic neighbor embedding (t-SNE) was displayed in Figure S5B. The heatmap showed higher expression levels of the seven risk-related m^5^C RNA methylation regulators (NOP2, DNMT3B, NSUN3, NSUN5, TET2, NSUN2, and NSUN4) in the high-risk group compared to the low-risk group (Figure 6(g)). The distributions of the risk scores and their survival status were displayed (Figure 6(h)). These results, taken together, suggested that this risk score was a good predictor for the prognosis of ccRCC.

### 3.7. m^5^C Risk Signature Can Be an Independent Prognostic Factor

So as to explore whether the risk signature can act as an independent prognostic factor, the univariate and multivariate Cox regression analyses were performed in the training cohort. The results indicated that the risk score was significantly related to the worse OS with HR = 2.796 (*p* < 0.001, 95% CI 1.977−3.954) (Table 4). Moreover, grade (HR = 2.242, 95% CI 1.682−2.988, *p* < 0.001), stage (HR = 1.862, 95% CI 1.541-2.251, *p* < 0.001), T stage (HR = 1.943, 95% CI 1.538-2.456, *p* < 0.001), M stage (HR = 4.073, 95% CI 2.634-6.300, *p* < 0.001), and N stage (HR = 2.932, 95% CI 1.516-5.668, *p* < 0.001) were also significantly associated with the OS according to the univariate Cox analysis (Table 4). In addition, the risk score was also related to the worse OS with HR = 1.779 (*p* = 0.002, 95% CI 1.594−2.147) according to the multivariate Cox analysis (Table 4). In conclusion, these results above suggested that signature-based risk score can be an independent prognostic factor for ccRCC.

### 3.8. Validation of the Risk Signature in the Testing Cohort and Entire Cohort

**Figure 7:**
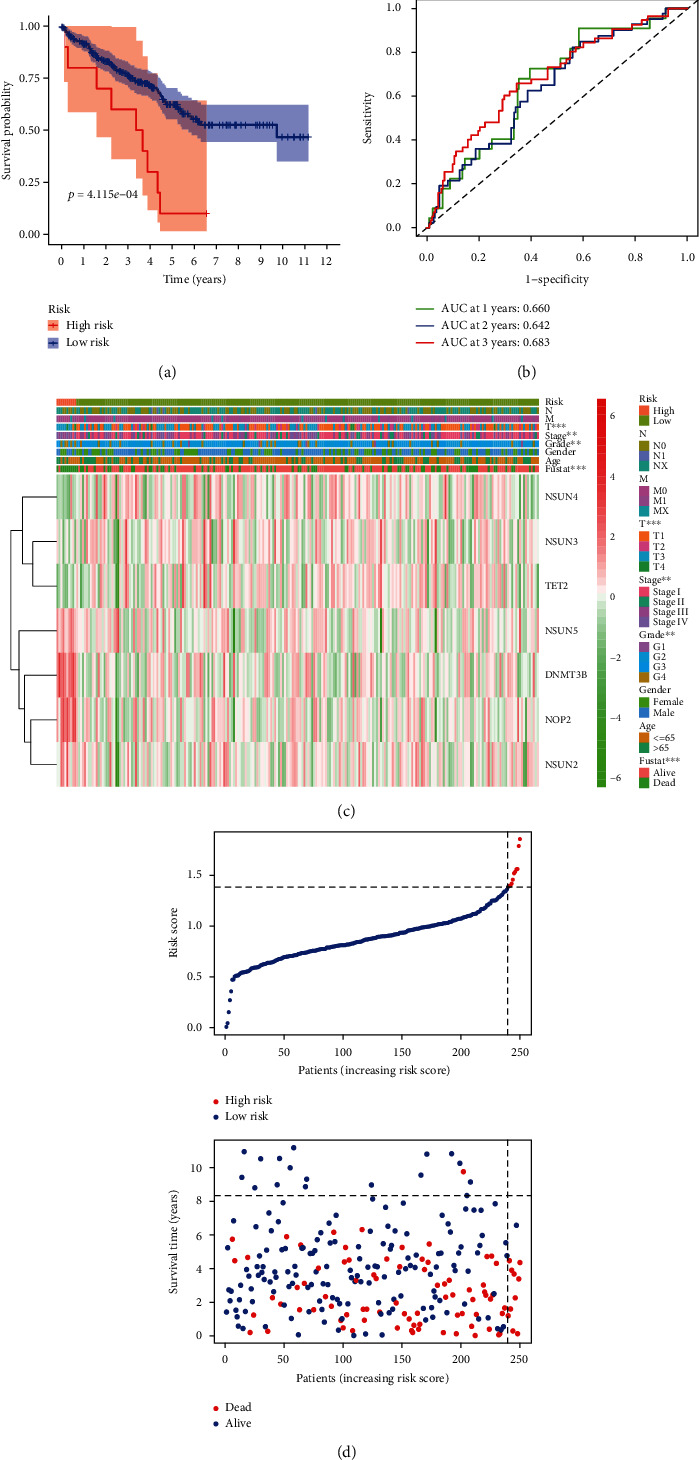
Validation of the prognostic risk signature in the testing cohort. (a) Kaplan-Meier curve analysis shows the OS of high-risk (*n* = 10) and low-risk (*n* = 236) ccRCC patients in the testing cohort. (b) ROC curve analysis in the testing cohort shows the false positive rate vs. true positive rate plots based on the prognostic risk signature. The AUC values for 1-year (green), 2-year (blue), and 3-year (red) survival rates are also shown. (c) The expression heatmap of the seven prognostic-risk related m^5^C RNA methylation regulators in the high-risk (blue) and low-risk (pink) ccRCC patients of the testing cohort. ^∗∗^*p* < 0.01; ^∗∗∗^*p* < 0.001. (d) The distributions of risk scores of the high-risk (red) and low-risk (blue) ccRCC patients and corresponding survival time (the red dots represent the dead patients and the blue dots represent the alive patients) in the testing cohort.

**Figure 8:**
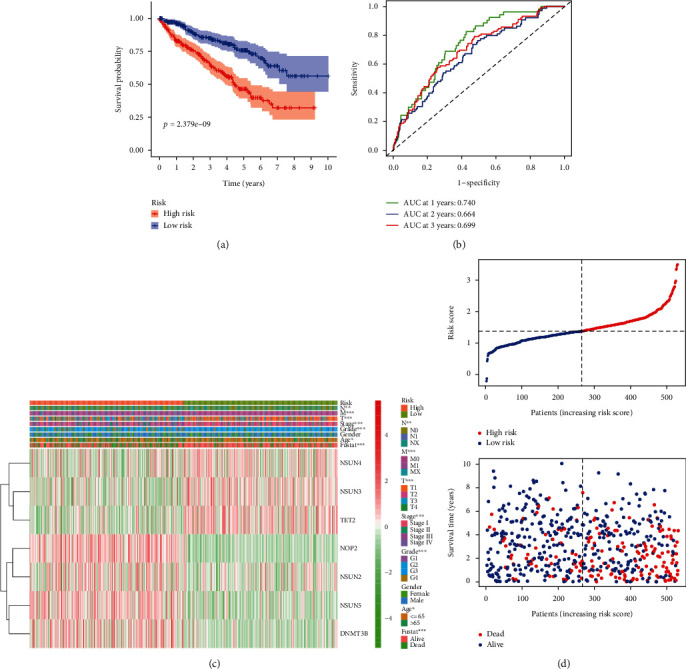
Validation of the prognostic risk signature in the whole TCGA-KIRC cohort. (a) Kaplan-Meier curve analysis shows the OS of high-risk (*n* = 258) and low-risk (*n* = 259) ccRCC patients in the entire TCGA cohort. (b) ROC curve analysis in the entire cohort shows the false positive rate vs. true positive rate plots based on the prognostic risk signature. The AUC values for 1-year (green), 2-year (blue), and 3-year (red) survival rates are also shown. (c) The expression heatmap of the seven prognostic-risk related m^5^C RNA methylation regulators in the high-risk (blue) and low-risk (pink) ccRCC patients of the entire cohort. ^∗∗^*p* < 0.01; ^∗∗∗^*p* < 0.001. (d) The distributions of risk scores of the high-risk (red) and low-risk (blue) ccRCC patients and corresponding survival time (the red dots represent the dead patients and the blue dots represent the alive patients) in the entire cohort.

To further verify the prognostic accuracy of the risk score, we tested it in the testing cohort and the whole cohort. The results of the univariate Cox regression analysis indicated that the risk score in the testing cohort, and the entire cohort was significantly related with the worse OS with HR = 5.245 (95% CI 1.792-15.355, *p* = 0.002) and HR = 2.796 (95% CI 1.977-3.954, *p* < 0.001), respectively (Table 4). Similarly, patients were divided into two groups according to the median risk score, respectively. Kaplan–Meier curves analysis in the testing cohort demonstrated that the patients in low risk had longer OS than those in high risk (Figure 7(a)). To detect the effectiveness of this model, the ROC analysis of 1/2/3-year OS was carried out in the testing cohort. The AUC values for the 1-, 2-, and 3-year survival in the testing cohort were 0.660, 0.642, and 0.683, respectively (Figure 7(b)). The results of PCA and t-SNE in the testing cohort were shown in Figure S5C and Figure S5D, respectively. The heatmap in the testing cohort showed that higher expression levels of the seven risk-related m^5^C RNA methylation regulators in the high-risk group compared to the low-risk group (Figure 7(c)). The distributions of the risk scores and the patient survival status between the low- and high-risk groups in the testing cohort were displayed in Figure 7(d). Similar results were further verified in the entire cohort. The result of Kaplan–Meier curves analysis in the entire cohort showed that the patients in low risk had longer OS than those in high risk (Figure 8(a)). The ROC values of 1-/2-/3-year OS in the entire cohort were 0.740, 0.664, and 0.699, respectively (Figure 8(b)). The result of PCA in the entire cohort was shown in Figure S5E. The result of t-SNE in the entire cohort was shown in Figure S5F. The heatmap in the entire cohort showed that these seven risk-related m^5^C RNA methylation regulators were highly expressed in the high-risk group compared to the low-risk group (Figure 8(c)). The distributions of the risk scores and the patient survival status between the low- and high-risk groups in the entire cohort were displayed (Figure 8(d)).

### 3.9. Construction of Nomogram in the Training Cohort, the Testing Cohort, and the Entire Cohort

**Figure 9:**
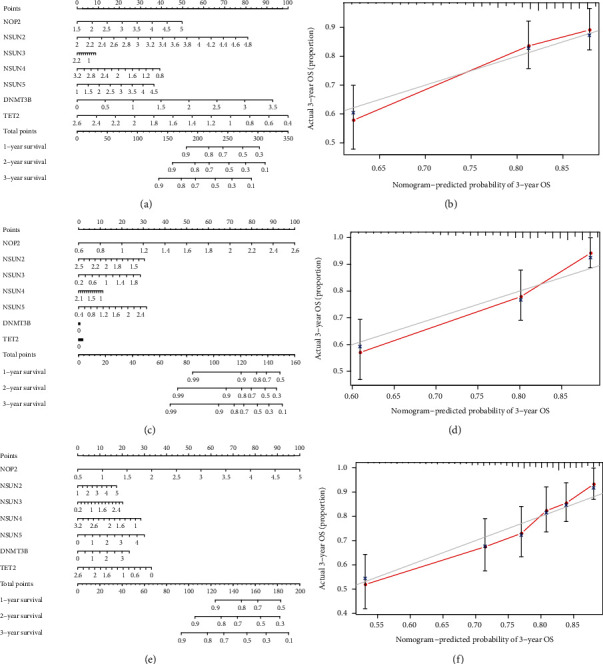
Nomograms to predict the survival rate of ccRCC patients in the training cohort, testing cohort, and the entire cohort. (a) The nomogram of used to predict the survival time, and (b) the calibration map used to predict the 3-year rate in the training cohort. (c) The nomogram used to predict the survival time, and (d) the calibration map used to predict the 3-year rate in the testing cohort. (e) The nomogram of used to predict the survival time, and (f) the calibration map used to predict the 3-year rate in the entire cohort.

In order to predict the 1-year, 2-year, and 3-year overall survival of each patient, the nomograms were designed in the training cohort, testing cohort and whole cohort, respectively. The expression signature for the seven risk-related genes was used as variables.  Figure 9(a) presented the seven variables of the training cohort, and the calibration curve compared well with the ideal model was shown in Figure 9(b). The nomograms for 1-, 2-, and 3-year OS were also constructed in the testing and entire cohort. The results in the testing cohort and the entire cohort were similar with that in the training cohort and were displayed in Figures 9(c) and 9(d) and Figures 9(e) and 9(f), respectively. These results above, taken together, suggested that m^5^C risk signature is a good predictor for the prognosis in ccRCC.

### 3.10. Kaplan-Meier Survival Curves of m^5^C RNA Methylation Regulators in ccRCC Patients

We further analyzed the association between the seven selected m^5^C RNA methylation regulator genes and the OS and disease-free survival (DFS) of ccRCC patients in the TCGA database. Kaplan-Meier survival curves and log-rank test showed that the OS of ccRCC patients with higher expression of DNMT3B, NOP2, NSUN2, and NSUN5 was significantly shorter compared to those with lower expression. In contrast, ccRCC patients with higher expression of NSUN3, NSUN4, and TET2 will have longer OS than those with lower expression (Figure S2A-G). Moreover, higher expression of NSUN3, NSUN4, and TET2 was correlated with significantly longer disease-free survival (DFS). However, there were any significant differences in the DFS of ccRCC patients with differential expression of NOP2, NSUN2, NSUN5, and DNMT3B (Figure S3A-G). Overall, our results demonstrated that these seven m^5^C RNA methylation regulators are potential prognostic biomarkers that can accurately predict survival outcomes of ccRCC patients.

### 3.11. GSEA Analysis Reveals Potential Signaling Pathways Related to Risk Score

**Figure 10:**
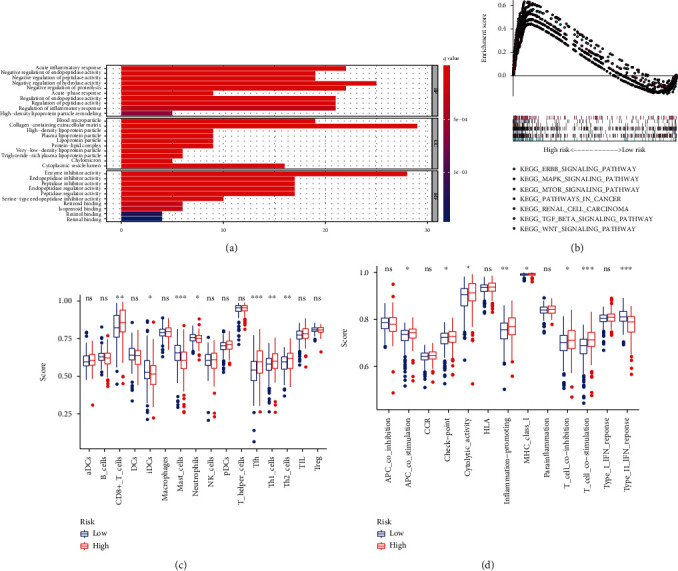
GSEA enrichment analysis. (a) The results of GO analysis in ccRCC with high-risk (red) and low-risk (blue) patients. (b) The seven most significantly enriched signaling pathways from KEGG. (c, d) Single-sample GSEA (ssGSEA) analysis showing the types of infiltrating immune cells (c) and the immune-related functions (d) in ccRCC with high risk (red) and low risk (blue). ^∗^*p* < 0.05, ^∗∗^*p* < 0.01, and ^∗∗∗^*p* < 0.001. ns: no significance.

To further understand the biological function and potential signaling pathways between the high-risk and low-risk groups, Gene Ontology (GO) and Kyoto Encyclopedia of Genes and Genomes (KEEG) pathway analyses were performed. The results of GO analysis indicated that the high-risk group are enriched in the regulation of various enzyme activities, such as negative regulation of hydrolase activity, negative regulation of proteolysis and so on (Figure 10(a)). Moreover, KEGG pathway analysis suggested that these genes were associated with various cancer-related pathways, including ERBB pathway, MAPK pathway, mTOR pathway, renal cell carcinoma, pathway in cancer, TGF-*β* pathway, and Wnt pathway, which gives a clue of the underlying mechanism in the pathogenesis of ccRCC (Figure 10(b) and S4, Table 5). Further, ssGSEA analysis was applied to explore the different infiltration degrees of immune cell types, immune-related functions, and immune-related pathways between the low-risk group and the high-risk group. The results indicated that the immune cells with different infiltration degrees between the two groups were T cells including follicular helper T cells, Th1 cells, Th2 cells, and CD8+ T cells (Figure 10(c)). The main differences in immune-related functions between the two groups were the interferon response and T cell costimulation (Figure 10(d)). The above results revealed that these seven selected genes were tumor-related, and the risk score can independently predict prognosis in patients with ccRCC.

### 3.12. Verification of Seven m^5^C RNA Methylation Regulators in Tissue Samples and Cell Lines

**Figure 11:**
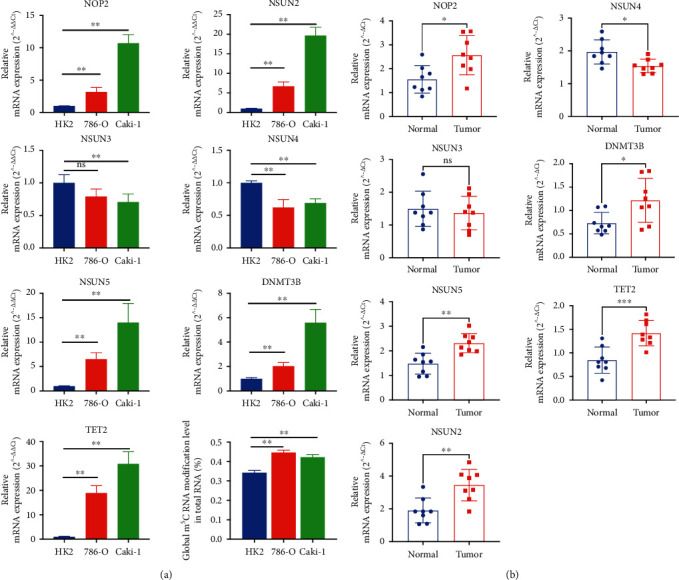
Verification of seven m^5^C RNA methylation-related genes in vitro and tissue samples. qRT-PCR experiment verified in renal cancer cell lines (a) and ccRCC tissue samples (b). NOP2, NSUN2, NSUN5, DNMT3B, and TET2 were significantly upregulated in cell lines and tissue samples, while NSUN4 was downregulated. However, NSUN3 mRNA expression level showed no significant difference. m^5^C RNA modification levels in the cell lines were measured by Global RNA Methylation Assay Kit (A). The bar graphs represent means ± standard deviation. ^∗∗^*p* < 0.05.

To further verify seven selected m^5^C RNA methylation-related genes' mRNA expression pattern in ccRCC, we performed qRT-PCR experiment in ccRCC tissue samples and cell lines. Among these m^5^C RNA methylation genes, we observed the significantly upregulated expression level of NOP2, NSUN2, NSUN5, DNMT3B, and TET2 in the renal cancer cell lines (786-O, Caki-1) compared with human renal tubular epithelial immortalized cell line (HK-2) (^∗∗^*p* < 0.01), while NSUN4 was downregulated in renal cell lines. However, NSUN3 mRNA expression level showed no significant difference (Figure 11(a)). Furthermore, we explored these m^5^C RNA methylation-related genes' expression level in clinical tissue samples, which was consistent with our results in the TCGA database and cell lines (Figure 11(b)). In addition, m^5^C RNA modification levels in the cell lines were measured by Global RNA Methylation Assay Kit. m^5^C RNA modification levels in renal cancer cell lines (786-O, Caki-1) were dramatically higher than that in HK-2 cell lines (Figure 11(a)).

## 4. Discussion

Renal clear cell carcinoma (ccRCC), the most common type of adult kidney carcinoma, is characterized by poor prognosis and high risk of metastasis and recurrence [17]. No effective therapeutic strategies for ccRCC patients with advanced stage or metastasis were founded, and the rate of 5-years disease-free survival in patients with metastasis is only 12% [18]. Therefore, identifying the effective diagnostic and prognostic biomarkers for early diagnosis and accurate prognosis is urgent to improve and prognose survival outcomes of ccRCC patients. The malignant progression and prognosis of ccRCC were associated with epigenetic modifications, including DNA methylation [19], histone modification [20], microRNA changes [21, 22], and RNA modification [23, 24]. The m^5^C methylation, the second common methylation modification, plays essential roles in various cellular, biological, and pathological processes. The methylation of cytosine is regulated by the genes that we call “writers,” “erasers,” and “readers.” The “writers” can upregulate m^5^C methylation, and the “erasers” can reverse the level of m^5^C methylation. The “readers” can bind to an m^5^C site and modulate differential biological signals.

Recent research revealed that m^5^C methylation is associated with prognosis in many cancers [9, 16]. The molecular mechanisms associated with the prognosis of ccRCC are still unknown. Therefore, we explored the relationship between m^5^C methylation regulators and the prognosis of ccRCC in this study.

In the present study, we found that eleven of twelve m^5^C RNA methylation regulators were abnormally expressed in ccRCC, among which, ten genes were associated with the prognosis. According to these ten selected m^5^C RNA methylation regulators, two clusters with significant differences of OS were distinguished in the ccRCC patients of TCGA. Moreover, on the basis of the training cohort, seven genes (NOP2, NSUN2, NSUN3, NSUN4, NSUN5, TET2, and DNMT3B) were screened for the construction of a prognostic risk signature. The results showed that the risk score was effective in predicting the clinical outcomes of ccRCC. Similarly, the independent prognostic value of this seven-gene risk score was represented again in the testing and the entire cohort, indicating that the risk signature had good performance in prognosis prediction. Univariate and multivariate Cox regression analyses in the whole cohort showed that age, stage, grade, TNM stage, and risk score were significantly associated with OS. the risk score could also predict the prognosis of the ccRCC patients with different clinicopathological parameters. The above results, taken together, suggested that the clinical outcomes were worse in the high-risk ccRCC patients than in the low-risk.

In our study, the higher expression level of NSUN4, NSUN3, and TET2 presented a better prognosis in ccRCC patients, indicating that these three genes might inhibit the progression of ccRCC. NSUN4 is a dual functional mitochondrial protein, enabling 12S rRNA methylation and coordinating mitochondrial assembly [25]. NSUN4 participates in cell proliferation and differentiation, protein biosynthesis, and cancer [26]. He et al. reported a high expression of NSUN4 in advanced liver cancer [27], contrary to our results. The reason may be that advanced tumors require a lot of energy and tumor cells improve their mitochondrial activity and obtain more energy by self-upregulating NSUN4 and other genes. NSUN3 was a putative methyltransferase in mitochondria [28], whose aberration may lead to a variety of diseases [29, 30]. Many studies have reported that TET2 can inhibit tumors, and its mutation can induce the occurrence and development of tumors [31–34]. Our results are consistent with the above results, indicating that TET2 is a tumor suppressor gene.

In contrast, the higher expression level of the other genes NOP2, DNMT3B, NSUN2, and NSUN5, the worse prognosis of ccRCC patients may have, which indicated that these four genes might promote the development of ccRCC. The high expression of NSUN5 can promote the proliferation of colon cancer cells [35], and the high expression of NOP2 can promote the metastasis of prostate cancer and the proliferation of liver cancer cells [36, 37]. We came to the same conclusion about NSUN5 and NOP2 genes in our study. DNMT3B is expressed as an oncogene in various of tumors, including leukemia, liver cancer, and bladder cancers [38–40]. Several studies have reported that NSUN2 can promote the development and metastasis of tumors [41–43].

To further understand the biological function and potential signaling pathways of these genes, we performed GO, KEGG, and ssGSEA analyses between tissues with different risk scores. The results above indicated that patients in the high-risk group might have the following changes in their bodies that result in a poor prognosis. Firstly, many articles have reported recently that the occurrence and development of tumors are related to inflammatory stimulation. Patients in the high-risk group may experience large inflammatory responses, resulting in more inflammatory cell infiltration and ultimately the inhibition of several key enzyme activities, which can induce cell apoptosis and mutation [44–46]. Secondly, the functions of various T cells including follicular helper T cells, Th1 cells, Th2 cells, and CD8+ T cells were suppressed in the high-risk group, causing the tumor cells to escape immunity and resulting in a poorer prognosis [47–49]. Thirdly, various cancer-related pathways, including ERBB pathway, MAPK pathway, mTOR pathway, pathways in cancer, TGF-*β* pathway, and Wnt pathway, were more likely to be activated, leading to poor outcomes. The mRNA expression level of m^5^C RNA methylation regulators and the global m^5^C RNA methylation level were measured in vitro and tissue samples, which is consistent with our above in silico analysis.

Some limitations of this study are noteworthy. The results of the current study, obtained by bioinformatics analysis, were not entirely accurate so that more experimental and clinical studies were still needed to further verify our results and find out the potential mechanism of m^5^C RNA methylation in ccRCC.

## 5. Conclusions

Our results demonstrated that eleven out of twelve m^5^C RNA methylation regulators are dysregulated between ccRCC tissues and normal tissues, among which ten genes were associated with prognosis. We defined m^5^C molecular subtypes and constructed a m^5^C methylation-related risk signature in the training cohort, by which the OS rate of ccRCC patients can be forecasted independently. The efficiency of the risk signature was further proved in the testing and whole cohort, respectively. The overall survival rate of patients with high risk may be lower. In addition, we found out the potential pathways of m^5^C RNA methylation-related genes in ccRCC and verified our results in vitro and clinical tissue samples.

## Figures and Tables

**Table 1:** Clinicopathological characteristics of patients in the TCGA-KIRC cohort.

Characteristic	Training cohort (*n* = 267)		Testing cohort (*n* = 246)		Entire cohort (*n* = 513)	
	Number	%	Number	%	Number	%
Age (years)						
≤65	174	65.2	164	66.7	338	65.9
>65	93	34.8	82	33.3	175	34.1
Gender						
Male	185	69.3	151	61.4	336	65.5
Female	82	30.7	95	38.6	177	34.5
Pathological stage						
Stage I	137	51.3	118	48.0	255	49.7
Stage II	30	11.2	24	9.8	54	10.5
Stage III	58	21.8	64	26.0	122	23.8
Stage IV	42	15.7	40	16.2	82	16.0
Histological grade						
G1	8	3.0	5	2.0	13	2.5
G2	109	40.8	114	46.3	223	43.5
G3	110	41.2	94	28.2	204	39.8
G4	40	15.0	33	13.5	73	14.2
T stage						
T1	141	52.8	120	48.8	261	50.9
T2	38	14.2	28	11.4	66	12.9
T3	83	31.1	92	37.4	175	34.1
T4	5	1.9	6	2.4	11	2.1
N stage						
N0	122	45.7	112	45.5	234	45.6
N1	9	3.4	5	2.0	14	2.7
Nx	136	50.9	129	45.5	265	51.7
M stage						
M0	214	80.1	196	79.7	410	79.9
M1	40	15.0	37	15.0	77	15.0
Mx	13	4.9	13	5.3	26	5.1
Outcome						
Alive	189	70.8	162	65.9	351	68.4
Dead	78	29.2	84	34.1	162	31.6

**Table 2:** Clinicopathological features between two clusters.

Characteristic	Cluster 1, *n* (%)	Cluster 2, *n* (%)	*p* value
Age (years)			
≤65	304 (66.5)	35 (58.3)	0.210
>65	153 (33.5)	25 (41.7)	
Gender			
Male	299 (65.4)	38 (63.3)	0.749
Female	158 (34.6)	22 (36.7)	
Pathological stage			
Stage I	234 (51.2)	25 (41.6)	0.003^∗∗^
Stage II	47 (10.3)	7 (11.7)	
Stage III	113 (24.7)	9 (15.0)	
Stage IV	63 (13.8)	19 (31.7)	
Histological grade			
G1	12 (2.6)	2 (3.3)	<0.001^∗∗∗^
G2	205 (44.9)	19 (31.7)	
G3	187 (40.9)	18 (30.0)	
G4	53 (11.6)	21 (35.0)	
T stage			
T1	240 (52.4)	25 (41.7)	<0.001^∗∗∗^
T2	56 (12.3)	10 (16.7)	
T3	158 (34.6)	17 (28.3)	
T4	3 (0.7)	8 (13.3)	
N stage			
N0	211 (46.1)	24 (40.0)	0.001^∗∗^
N1	8 (1.8)	6 (10.0)	
Nx	238 (52.1)	30 (50.0)	
M stage			
M0	376 (82.3)	36 (60.0)	<0.001^∗∗∗^
M1	60 (13.1)	17 (28.3)	
Mx	21 (4.6)	7 (11.7)	
Outcome			
Alive	320 (70.0)	33 (55.0)	0.019^∗^
Dead	137 (30.0)	27 (45.0)	
Total			
517	457	60	

^∗^ *p* < 0.05, ^∗∗^*p* < 0.01, and ^∗∗∗^*p* < 0.001.

**Table 3:** List of m^5^C RNA methylation-related genes for constructing prognostic risk score.

Genes	Types	Coefficient	Univariate Cox analysis		
			HR	95% CI	*p* value
NOP2	m^5^C writer	0.3587	2.701	2.097-3.480	<0.001
NSUN2	m^5^C writer	0.2297	1.812	1.185-2.771	0.006
NSUN3	m^5^C writer	-0.0095	0.567	0.332-0.969	0.038
NSUN4	m^5^C writer	-0.0992	0.547	0.344-0.871	0.011
NSUN5	m^5^C writer	0.1508	2.333	1.714-3.176	<0.001
DNMT3B	m^5^C writer	0.4538	2.039	1.557-2.670	<0.001
TET2	m^5^C eraser	-0.6042	0.531	0.366-0.770	<0.001

HR: hazard ratio, estimated from Cox proportional hazard regression model; CI: confidence interval of the estimated HR.

**Table 4:** Univariate and multivariate Cox regression analyses of the TCGA cohort for risk score of m^5^C-related genes.

Variable	Univariate Cox regression			Multivariate Cox regression		
	HR	95% CI	*p* value	HR	95% CI	*p* value
Age	1.023	1.005-1.041	0.012	1.033	1.013-1.054	0.001
Gender	0.951	0.666-1.541	0.951	1.205	0.769-1.887	0.416
Grade	2.242	1.682-2.988	<0.001	1.529	1.088-2.149	0.014
Stage	1.862	1.541-2.251	<0.001	1.435	0.843-2.441	0.183
T	1.943	1.538-2.456	<0.001	1.010	0.618-1.651	0.969
M	4.073	2.634-6.300	<0.001	1.714	0.751-3.912	0.201
N	2.932	1.516-5.668	0.001	1.593	0.768-3.304	0.211
Risk score	2.796	1.977-3.954	<0.001	1.779	1.594-2.147	0.002

HR: hazard ratio, estimated from Cox proportional hazard regression model; CI: confidence interval of the estimated HR.

**Table 5:** Gene set enrichment analysis result of the high-risk group.

Gene set name	NES	NOM p-value	FDR q-value
KEGG renal cell carcinoma	2.21	0.000	0.017
KEGG TGF beta signaling pathway	2.19	0.002	0.016
KEGG WNT signaling pathway	2.15	0.000	0.013
KEGG ERBB signaling pathway	2.11	0.000	0.011
KEGG pathways in cancer	2.02	0.006	0.015
KEGG MAPK signaling pathway	1.98	0.008	0.017
KEGG mTOR signaling pathway	1.98	0.004	0.017

NES: normalized enrichment score; NOM: nominal, FDR: false discovery rate. Gene sets with NOM *p* value < 0.01 and FDR *q* value < 0.02 are considered significant.

## Data Availability

The data which support the results of this study are available in databases described in the manuscript and from the corresponding authors upon request.

## Supplementary Materials

Supplementary Materials Figure S1: the expression levels of m^5^C regulatory genes in tumors. (A) The heat map of twelve m^5^C-related genes in various tumors obtained from the TCGA database. (B–H) The expression levels of the seven prognostic-risk signature genes in various cancers in the TCGA database with the corresponding high or low expression. (B) NOP2. (C) NSUN4. (D) NSUN3. (E) NSUN2. (F) DNMT3B. (G) TET2. (H) NSUN5. Figure S2: Overall survival (OS) from the TCGA-KIRC database with high or low expression of the seven prognostic-risk signature genes by Kaplan-Meier survival curve. Overall survival analyses of seven prognostic-risk signature genes in the TCGA-KIRC cohort by Kaplan-Meier with a log-rank test. (A) NOP2. (B) NSUN2. (C) NSUN3. (D) NSUN4. (E) NSUN5. (F) TET2. (G) DNMT3B. Figure S3: disease-free survival (DFS) from the TCGA-KIRC database with the high or low expression of the seven prognostic risk signature genes by Kaplan-Meier survival curve. Disease-free survival analyses of seven prognostic-risk signature genes in the TCGA-KIRC cohort by Kaplan-Meier with a log-rank test. (A) NOP2. (B) NSUN2. (C) NSUN3. (D) NSUN4. (E) NSUN5. (F) TET2. (G) DNMT3B. Figure S4: KEGG pathways enrichment plot. (A) TGF-*β* signaling pathway. (B) Renal cell carcinoma. (C) Wnt signaling pathway. (D) ERBB signaling pathway. (E) mTOR signaling pathway. (F) Pathways in cancer. Figure S5: the analyses of PCA and t-SNE between the high-risk and low-risk patients in the training cohort, testing cohort, and entire cohort, respectively. (A, B) The results of PCA (A) and t-SNE (B) between the high-risk (red) patients and the low-risk (blue) patients in the training cohort. (C, D) The results of PCA (C) and t-SNE (D) between the high-risk (red) patients and the low-risk (blue) patients in the testing cohort. (E, F) The results of PCA (E) and t-SNE (F) between the high-risk (red) patients and the low-risk (blue) patients in the entire cohort. Table S1: oligonucleotide sequences used in this study.
