# Supplementary material for: Comprehensive Analysis of m5C RNA Methylation Regulator Genes in Clear Cell Renal Cell Carcinoma
Source: Int J Genomics. 2021 Sep 28;2021:3803724. doi: 10.1155/2021/3803724 (PMC8497170; doi:10.1155/2021/3803724)
Supplement: Supplementary Materials — Figure S1: the expression levels of m5C regulatory genes in tumors. (A) The heat map of twelve m5C-related genes in various tumors obtained from the TCGA database. (B–H) The expression levels of the seven prognostic-risk signature genes in various cancers in the TCGA database with the corresponding high or low expression. (B) NOP2. (C) NSUN4. (D) NSUN3. (E) NSUN2. (F) DNMT3B. (G) TET2. (H) NSUN5. Figure S2: Overall survival (OS) from the TCGA-KIRC database with high or low expression of the seven prognostic-risk signature genes by Kaplan-Meier survival curve. Overall survival analyses of seven prognostic-risk signature genes in the TCGA-KIRC cohort by Kaplan-Meier with a log-rank test. (A) NOP2. (B) NSUN2. (C) NSUN3. (D) NSUN4. (E) NSUN5. (F) TET2. (G) DNMT3B. Figure S3: disease-free survival (DFS) from the TCGA-KIRC database with the high or low expression of the seven prognostic risk signature genes by Kaplan-Meier survival curve. Disease-free survival analyses of seven prognostic-risk signature genes in the TCGA-KIRC cohort by Kaplan-Meier with a log-rank test. (A) NOP2. (B) NSUN2. (C) NSUN3. (D) NSUN4. (E) NSUN5. (F) TET2. (G) DNMT3B. Figure S4: KEGG pathways enrichment plot. (A) TGF-β signaling pathway. (B) Renal cell carcinoma. (C) Wnt signaling pathway. (D) ERBB signaling pathway. (E) mTOR signaling pathway. (F) Pathways in cancer. Figure S5: the analyses of PCA and t-SNE between the high-risk and low-risk patients in the training cohort, testing cohort, and entire cohort, respectively. (A, B) The results of PCA (A) and t-SNE (B) between the high-risk (red) patients and the low-risk (blue) patients in the training cohort. (C, D) The results of PCA (C) and t-SNE (D) between the high-risk (red) patients and the low-risk (blue) patients in the testing cohort. (E, F) The results of PCA (E) and t-SNE (F) between the high-risk (red) patients and the low-risk (blue) patients in the entire cohort. Table S1: oligonucleotide sequences used in this study. [file 3803724.f1.zip › Table S1.docx]

**Table S1 Oligonucleotide sequences used in this study**

| Primers | Sequences | |
| --- | --- | --- |
| NOP2 | Forward | AAGGGTGCCGAGACAGAACT |
|  | Reverse | GAGCACGACTAGACAGCCTC |
| NSUN2 | Forward | CAAGCTGTTCGAGCACTACTAC |
|  | Reverse | CTCCCTGAGAGCGTCCATGA |
| NSUN3 | Forward | CATGCTGGCAATATGCTGTCC |
|  | Reverse | AAAGATCCCTGAGAGAGTGTGT |
| NSUN4 | Forward | CCATCAATCCGTGTCAGTCTC |
|  | Reverse | GCTTAGCACTTACATGATCCCAG |
| NSUN5 | Forward | CGCTACCATGAGGTCCACTAC |
|  | Reverse | GCATCTCGCACCACGTCTT |
| DNMT3B | Forward | AGGGAAGACTCGATCCTCGTC |
|  | Reverse | GTGTGTAGCTTAGCAGACTGG |
| TET2 | Forward | GATAGAACCAACCATGTTGAGGG |
|  | Reverse | TGGAGCTTTGTAGCCAGAGGT |
| β-actin | Forward | ATGACTTAGTTGCGTTACACC |
|  | Reverse | GACTTCCTGTAACAACGCATC |
